# Can we use the ankle non-invasive blood pressure during otolaryngologic surgery: an observational study

**DOI:** 10.11604/pamj.2020.36.31.21019

**Published:** 2020-05-21

**Authors:** Sidi Driss El Jaouhari, Mohamed Meziane, Jalal Kessouati, Rachid Razine, Abdelhamid Jaafari, Mustapha Bensghir

**Affiliations:** 1 Department of Anesthesiology and Critical Care, Faculty of Medicine and Pharmacy, Military Hospital Mohamed V, University of Mohamed V Souissi, Rabat, Morocco; 2 Laboratory of Epidemiology and Clinical Research, Faculty of Medicine and Pharmacy, University of Mohamed V Souissi, Rabat, Morocco

**Keywords:** Ankle blood pressure, brachial blood pressure, otolaryngologic surgery, controlled hypotension

## Abstract

**Introduction:**

In otolaryngologic surgery, ankle is frequently used for monitoring anesthesia in place of brachial when the patient doesn´t need invasive arterial cannulation. If there is a clinically useful and Predictable link between the two readings in hemodynamic normal patient, this difference during otolaryngologic surgery, was not evaluated. We aimed to investigate the reliability and the acceptability of non invasive blood pressure measurements at the ankle compared to those obtained concurrently at the arm during otolaryngologic surgery.

**Methods:**

Eighty ASA grade I and II patients who had to be operated under general anesthesia were taken as subjects for our study. Blood pressures were measured simultaneously in the 2 limbs before induction and then every 10 minutes until the end of the surgical procedure. Readings were initiated concurrently. Statistical analysis was performed with PASW Statistics 13.

**Results:**

There were 41 males (51.2 %) and 39 females (48.8 %). Bland-Altman analysis of mean difference between the ankle and arm (95 % limits of agreement) was -11.47 (- 23.77 to 0.82) mmHg for systolic blood pressure (SBP), -7.89 (-19.16 to 3.36) mmHg for diastolic blood pressure (DBP) and - 9.09 (18.19 to 0.00) mmHg for mean arterial pressure (MAP). Non-parametric analysis showed that 67.5 % of SBP, 46.2 % of DBP and 56.2 % of MAP measurements differed by > 10mmHg.

**Conclusion:**

Ankle BP cannot be used routinely in otolaryngological surgery. Although, the ankle can be used as an alternative where the arm cannot be used taking into account a difference.

## Introduction

Good surgical field visibility is one of the basic prerequisites for a precise and safe otolaryngologic surgery and the main obstacle to good visibility is excessive perioperative bleeding. Studies have shown a variety of methods reducing bleeding in the surgical field through: lowering the mean arterial pressure (MAP), lowering heart rate (HR), local anemization with adrenaline, preoperative use of steroids and reversing the trendelenburg position, which reduces blood supply in the surgical field [1]. Controlled hypotension is a common procedure during anesthesia applied to patients undergoing otolaryngologic surgery and especially endoscopic sinus intervention. Decreased blood pressure (BP) allows reduction of bleeding in the surgical field, minimization of blood loss, better visibility and therefore, it increases the surgeon´s comfort, reduces the surgery time and prevents complications emerging from blurred vision caused by coverage of the camera lens with blood [2,3]. In otolaryngologic surgery, ankle BP is frequently used for monitoring anesthesia in place of brachial BP when the patient doesn´t need invasive arterial cannulation.

Given the particularity of cephalic surgery and the difficult access to the head and upper limb, ankle BP allows a better maneuverability for the anesthetists. Lack of ankle-arm BP differences, can lead to error in the anesthetic management. Wax *et al.* have shown in their study that inadequate BP monitoring is associated with inadequate intraoperative blood transfusions, vasopressor infusions and antihypertensive medication administration [4]. Due to the lack of manuscripts that statistically analyzes arm-ankle BP differences during otolaryngologic operation and given the potential benefits of blood pressure measurements at the ankle, we aimed to investigate the reliability and the acceptability of non-invasive blood pressure (NIBP) measurements at the ankle compared to those obtained concurrently at the arm during otolaryngologic surgery.

## Methods

Our study was approved by our Institutional Ethics Committee over a period of two months (April and May 2019). After obtaining written informed consent from all participants, eighty ASA grade I and II patients who had to be operated under general anesthesia were taken as subjects for our study. Patients with significant cardiovascular pathology, hypertension, with BP above 160/110 mmHg, musculoskeletal abnormalities, pregnant women, child under 14 and patients known to have peripheral arterial disease were excluded. After the patient was shifted to the operating theater, he was asked to relax for five minutes. The circumferences of the left arm immediately proximal to the antecubital fossa and the left ankle immediately above the malleolus were used to select appropriately sized NIBP cuffs. The cuff bladder was sited anteriorly on the arm and posteriorly on the ankle. The standard position was made with the arm resting on the operation theatre table, at the level of the heart. Blood pressures were measured simultaneously in the two limbs with a GE healthcare carescape V100 vital signs monitor before induction and then every 10 minutes until the end of the surgical procedure.

Systolic blood pressure (SBP), diastolic blood pressure (DBP) and mean arterial pressure (MAP) were recorded by a nurse single researcher, who did not have clinical responsibility for the case. Readings were initiated concurrently. Any readings that took a prolonged period to measure were noted. Drugs used for induction of anesthesia were the same in whole patients (fentanyl: 5μ/kg, propofol: 3mg/kg and esmeron: 0.5mg/kg). The attending anesthetist was blinded to the ankle blood pressure readings. In addition to the registration of the BP values, the following data were collected: demographic characteristics, medical background, ASA physical status, type of otolaryngologic operation, the need for deepening anesthesia, the wake-up delay and the existence of a wake-up incident. The paired t-test was used for comparisons of paired parametric data and the McNemar test for paired categorical data. The t-test was used for unpaired parametric data and Fisher´s exact test for unpaired categorical data. Modified Bland-Altman analysis partitioning between - and within - patient agreement was used to compare arm and ankle blood pressure measurements [5]. Statistical analysis was performed with PASW Statistics 18.0 (Predictive Analytics Software; SPSS Inc., Chicago, Illinois, USA).

## Results

**Figure 1:**
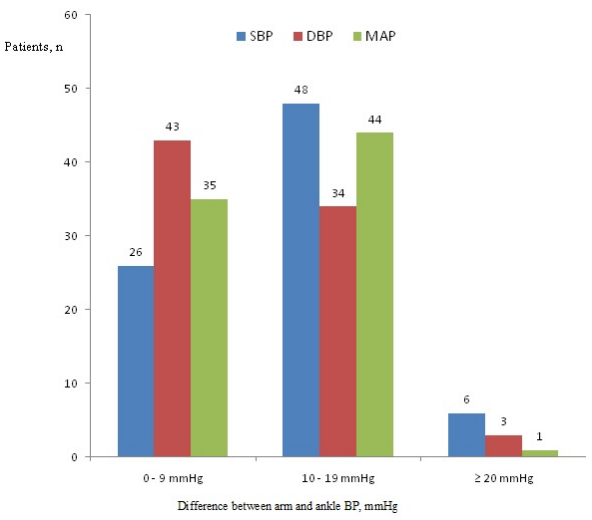
Error categories of absolute differences in systolic, diastolic blood pressure and mean arterial pressure between arm and ankle measurements

**Figure 2:**
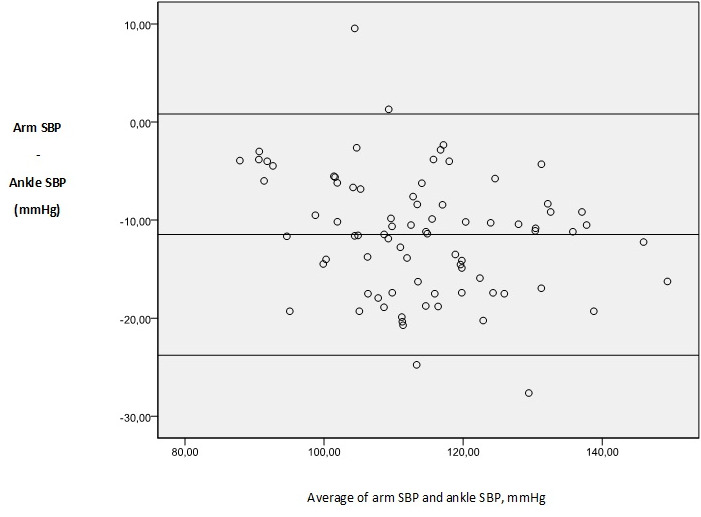
Bland-Altman plots of arm v. ankle systolic blood pressure

**Figure 3:**
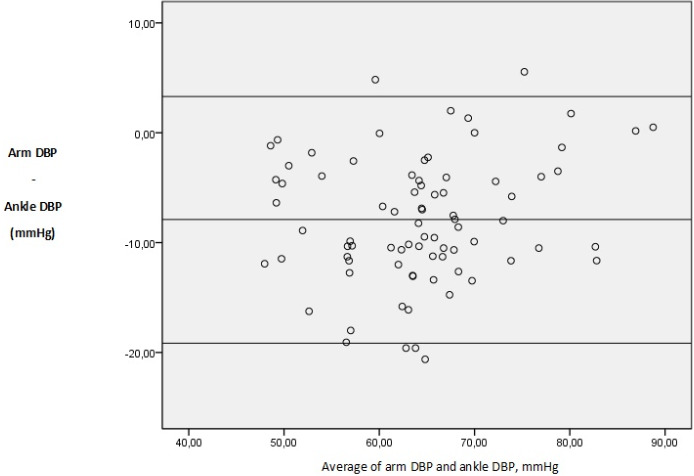
Bland-Altman plots of arm v. ankle diastolic blood pressure

**Figure 4:**
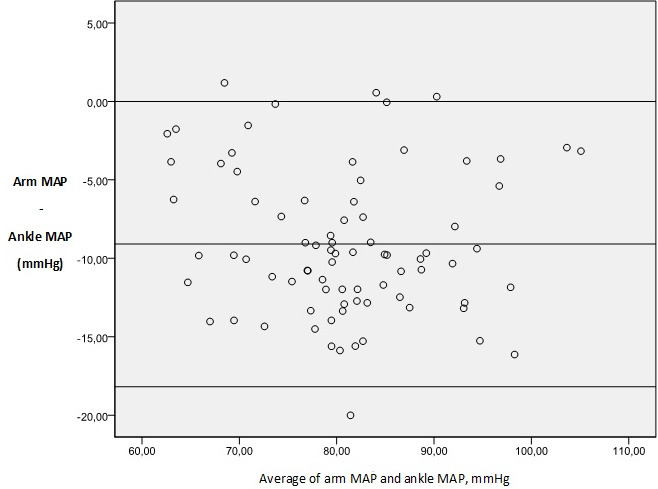
Bland-Altman plots of arm v. ankle mean arterial pressure

Eighty patient candidates for otolaryngologic elective surgery were enrolled into the study. The data on 80 patients were included for analysis. There were 41 males (51.2 %) and 39 females (48.8 %). The basic demographic data are summarized in Table 1. In 100% of the study population, a standard adult-sized appropriate cuff allowed all the measures for both the patient´s arm and ankle. There was a clinically (i.e. a difference of ≥ 10mmHg) and statistically significant difference between arm and ankle SBP. There was a statistically but not clinically significant difference between arm and ankle DBP and MAP (Table 2). Difference comparison between the arm and the ankle BPs by category showed that 67.5 % of SBP, 46.2 % of DBP and 56.2 % of MAP measurements have a difference >10 mmHg (Figure 1). Results were analyzed comparing SBP, DBP and MAP at each of the two sites in 80 patients using the Bland-Altman approach. The limits of agreement were calculated by evaluating the difference between each pair of scores. Mean differences between pairs of reading (arm-ankle) was - 11.47mmHg for SBP, - 7.89mmHg for DBP and - 9.09mmHg for MAP, with 95 limits of agreements for single observations of - 23.77 to 0.82mmHg for SAP, - 19.16 to 3.36mmHg for DAP and - 18.19 to 0.00mmHg for MAP (Table 3, Figure 2, Figure 3, Figure 4 for the Bland-Altman graphic representation). Intraoperatively, the anesthesia of 58% of the patients was deepened at the request of surgeons, either by injecting drugs or by increasing the MAC or by combining the two. The average time between the end of anesthesia maintenance and extubation was 14.7 minutes. No per or postoperative incident was noted.

**Table 1:** Basic demographic data

	Total (N=80)	Males (n=41)	Females (n=39)
Age (years), mean±ET	43.31±14.35	45.85±12,85	40.64±15.49
BMI(Kg/m^2^), mean±ET	25.56±3.87	25.94±2.77	25.24±4.65
**ASA, n(%)**			
I	72(90%)	38(92.7%)	34(87.2%)
II	8(10%)	3(7.3%)	5(12.8%)

(E : Ecart type; BM : body masse index; ASA: American society of anesthesiology)

**Table 2:** Differences between mean arm and mean ankle BPs

	**BP Arm(mmHg)**	**BP Ankle(mmHg)**	**Arm-ankle, difference**	**p-value**
**SBP**	****	****	****	****
Male	109.9	121.32	-11.42	<0.0001
Female	106.6	118.16	-11.56	<0.0001
All	108.31	119.78	-11.47	<0.0001
**DBP**				****
Male	62.43	69.57	-7.14	<0.0001
Female	58.16	66.86	-8.7	<0.0001
All	60.35	68.25	-7.9	<0.0001
**MAP**	****	****	****	****
Male	78.27	86.82	-8.55	<0.0001
Female	74.31	83.96	-9.65	<0.0001
All	76.34	85.43	-9.09	<0.0001

BP: blood pressure, SBP: systolic blood pressure, DBP: diastolic blood pressure, MAP: mean arterial pressure

**Table 3:** Limits of agreement for SBP, DBP and MAP (mmHg)

	Mean difference	95% limits of agreement
SBP arm-ankle	-11.47	-23.77 to 0.82
DBP arm-ankle	-7.89	-19.16 to 3.36
MAP arm ankle	-9.09	-18.19 to 0.00

(SBP: systolic blood pressure, DBP: diastolic blood pressure, MAP: mean arterial pressure.)

## Discussion

As early as 1925, it was found that the systolic BP was 20 - 40 mmHg higher in the leg than in the arm in normal subjects at rest [6]. If there is a clinically useful and predictable link between the two readings in hemodynamic normal patient, this difference during otolaryngologic surgery, was not evaluated. The mean difference (arm - ankle) found in this survey, concerning the 3 types of measures (SBP, DBP and MBP), was found negative, with importance for SAP less for DAP and MAP. Although the average differences for MAP and DBP that they observed were below clinical significance, the limits of agreement were wide. In this study, there was a larger difference between arm and ankle SBP, with the BPs for both genders being outside the clinically acceptable range (i.e. a difference of ≥ 10mmHg). The mean SBP measured in the arm was 11.47mmHg lower than that measured in the ankle. This difference is not constant. The arm SBP was up to 0.82mmHg higher or conversely 23.77mmHg lower than the measured SBP in the arm (95% CI).

This shows that this difference is statistically and clinically significant and translates to a non-detection of significant variation of BP. Particularly when comparing SAP between the two sites of measurements, the degree of discrepancy in unacceptable for ankle NIBP to be used routinely. DBP fared best, with the mean difference between the arm and ankle being - 7.89mmHg. Although it seems very small, reading at opposite extremes of the 95% limits of agreement of arm and ankle could differ by as much as 22.52mmHg, with the actual CI range being -19.16; 3.36mmHg and only 53% of patients had a mean difference lower than 10mmHg. This shows that this difference is statistically significant and clinically not significant, within the clinically acceptable error range of 10mmHg. The MAP was also statistically significantly different between the arm and the ankle, with higher MAPs noted in the ankle. The MAP actual mean difference between the arm and the ankle fell within the clinically acceptable error range of 10mmHg but was 9mmHg higher than the mean arm MAP. Extreme of the 95% limits of agreement of arm and ankle could differ by as much as 18.19 for MAP.

Knowing that the MAP is the perfusion pressure of the organs, this indicates that in our study, the value of this pressure found on the ankle is always higher than that found at the arm. The basis of NIBP measurement by oscillometry is the determination of MAP from the maximum pulse oscillation during cuff deflation [7]. Systolic and diastolic pressure can only be estimated indirectly according to some empirically derived algorithm [8]. Variation in propagation of pressure waves as they pass down the descending aorta results in narrowing and increase in the systolic portions of the pressure wave in the ankle compared with the aortic arch, when measured directly in the anaesthetized dog [9]. That´s way the SBP is often higher in an ankle than the arm, with MAP remaining approximately similar throughout the arterial system. Overall, different studies have demonstrated that SBP measurements were higher in the calf than in the arm, particularly in patients undergoing surgery and colonoscopy [10,11]. Moore *et al.* [12] in a study comparing BP in the arm, calf and ankle concluded that there was a poor agreement between the different sites with respect to SBP.

The agreement was closer for DBP and MBP measurements. The difference in BP between the upper and lower limbs in pregnant women has been subject of several studies. Zahn *et al.*, [13] found that pregnant patients had significant differences in their SBP and DBP, but no differences in their MAP. Sanghera *et al.* compared arm and ankle blood pressure under spinal anesthesia for cesarean section [14] and found no association between arm and ankle BPs in pregnant patients during caesarean section. They found only marginal agreement between the two methods and concluded that the ankle was not a suitable alternative in this circumstance. Another study comparing NIBP measurement at the arm and ankle during caesarean section [15] concluded that measuring blood pressure is feasible at the ankle instead of at the arm and that the overall mean difference between paired readings of MAP and DAP is very small, however the lack of precision is too great to support routine use of ankle NIBP. Inconsistent results with arm and ankle BP measurements were also found in anesthetized children.

In children 8 years and younger, BPs were found to be lower in the leg than in the arm [16], whereas another study showed no link between arm and ankle BP [17]. In otolaryngologic surgery and especially in endoscopic transnasal surgery, intraoperative bleeding must be kept low. One of the main methods of reducing perioperative bleeding is the use of controlled hypotension [3]. Although the positive effects of controlled hypotension are well known, the concerns of severe hypotension limit the method. Permanent brain damage, emboli in cerebral circulation, difficult awakening of the patient, and even death - all these complications might occur when hypotension is too deep [2,18]. The influence of controlled hypotension during endoscopic procedures on middle cerebral artery peak systolic velocity was additionally evaluated [19]. More than half of patients operated standard flow rate fell below the lower limit of normal and end-diastolic velocity was below the limit of even 60% of patients in these conditions, as is apparent from earlier studies could be a risk of ischemic brain tissue.

Despite that, no neurological disturbances were found in any of the patients in the postoperative period. Also, in the study conducted by Stanistaw *et al.* concerning the impact of controlled induced hypotension on cognitive functions of patients undergoing functional endoscopic sinus surgery (FESS), they found that the degree of controlled intraoperative hypotension during FESS did not influence the results of psychometric tests as well as no complications in the functions of kidneys, lungs, nervous system and cardiovascular system were observed [1]. It seems to be equally safe for the patient as anesthetic management in normotension [1]. In our study, the measure of the ankle BP is lower than the arms BP, which suggests that the BP displayed is lower than the standards. Also, no postoperative incidents and no waking delay were noted. The choice of an adult sample ASA 1 and 2, devoid of cardiovascular disease makes it relative result because it is the association of risk factors which causes cognitive dysfunctions: type of surgery, duration and type of anesthesia administered during surgery, advanced patient age, history of alcohol abuse and use of anticholinergic medications [20].

## Conclusion

A comparison of NIBP in the arm and ankle in admitted patients to otolaryngologic surgery has shown that NIBP readings taken from the ankle tend to be higher when compared with those taken from the arm. The least significant difference found was the DBP, which unfortunately does not have many clinical applications as a target in the resuscitation setting. Given the great lack of precision and the wide limits of agreement, ankle BP cannot be used routinely especially in otolaryngologic surgery, whose hypotension controlled with the maintenance of good hemodynamic stability, is the basis of good anesthetic management. Although, the ankle can be used as an alternative site for measurement of BP where the arm cannot be used, taking into account a difference.

### What is known about this topic

- There is a difference in arterial pressure between the arm and ankle;
- Controlled hypotension is a common procedure during anesthesia applied to patients undergoing otolaryngologic surgery.

### What this study adds

- In otolaryngologic surgery ankle NIBP tend to be higher when compared with those taken from the arm;
- Ankle BP cannot be used routinely especially in otolaryngologic surgery.

## Competing interests

The authors declare no competing interests.
